# Local recurrence of phyllodes tumors after surgery with wide compared to narrow margins: study protocol for a systematic review and meta-analysis

**DOI:** 10.1186/s13643-025-02904-1

**Published:** 2025-07-08

**Authors:** Carl Sars, Jan Frisell, Paul W. Dickman, Helena Sackey, Ebba K. Lindqvist

**Affiliations:** 1 Department of Molecular Medicine and Surgery, Karolinska Institutet, Stockholm 171 77, Sweden; 2 Division of Cancer, Department of Breast, Endocrine Tumors and Sarcoma, Karolinska Comprehensive Cancer Center, Karolinska University Hospital, Stockholm, Sweden; 3 Department of Medical Epidemiology and Biostatistics, Karolinska Institutet, Stockholm, Sweden; 4 Department of Clinical Science and Education, Stockholm South General Hospital, Karolinska Institutet, Stockholm, Sweden; 5 Department of Surgery, Stockholm South General Hospital, Stockholm, Sweden

**Keywords:** Margins of excision, Systematic review, Meta-analysis, Phyllodes tumor, Neoplasm recurrence, Local

## Abstract

**Background:**

Phyllodes tumors are rare fibroepithelial breast lesions graded as benign, borderline, or malignant. Surgical resection with clear margins is the primary method of treatment. Consensus on margin width could prevent unnecessary large primary resections or reoperations, yet the optimal margins for different tumor grades remain unclear. This systematic review and meta-analysis will evaluate the effect of wide versus narrow margins on local recurrence rates after surgery for phyllodes tumors of the breast. In addition, the re-excision rates, adjuvant treatment and adverse events will be assessed.

**Methods:**

An information specialist will assist in searching MEDLINE, EMBASE, Web of Science, Google Scholar, and Cochrane Library databases, as well as gray literature to identify randomized controlled trials, observational studies, and case series. Relevant abstracts from professional society meetings and web-based registries of clinical trials will also be included via hand-search and by forward-tracking papers and by searching the reference lists of the obtained articles. Studies included will compare patients, without age limitation, who were surgically treated for a histopathologically confirmed phyllodes tumor of the breast. Studies reporting both local recurrence rate and surgical excision margins will be included. No language restriction will be applied. Two reviewers will independently screen the titles and abstracts of the studies identified during the search using pre-defined inclusion criteria and data extraction from the full texts of selected studies will be performed. The quality of included studies will be assessed by two independent reviewers using the Cochrane Risk of Bias 2.0 tool for randomized trials, the Newcastle–Ottawa Scale for observational studies and the Joanna Briggs Institute Checklist for case series. A meta-analysis on pooled local recurrence rates will be conducted, stratified by different phyllodes tumor grades and surgical margins.

**Discussion:**

This systematic review will provide a synthesis of current evidence on the optimal surgical margins of phyllodes tumors and its effect on local recurrence rates. These findings aim to provide clinicians with guidelines and to establish a strong research base for future studies in this field.

**Trial registration:**

PROSPERO CRD420250640098.

**Supplementary Information:**

The online version contains supplementary material available at 10.1186/s13643-025-02904-1.

## Background

Phyllodes tumors are rare fibroepithelial lesions of the breast that can be graded as benign, borderline, and malignant [1]. In addition to challenges with grading, benign phyllodes tumors share overlapping characteristics with fibroadenomas, a common benign breast neoplasm [2]. At the other end of the spectrum, malignant phyllodes tumors are difficult to distinguish from primary breast sarcomas [2]. Surgical resection is the primary method of treatment, and clear margins are considered essential to minimize the risk of local recurrence [3]. Determining the excision margins required is crucial, as there may be a trade‐off between a better cosmetic and functional result and poorer long‐term local control if margins become too narrow. Furthermore, reaching consensus on accepted margin widths could spare patients unnecessary secondary surgery to obtain a preferred wider margin [4, 5]. However, the optimal margin width for different grades of phyllodes tumors (benign, borderline, and malignant) remains a topic of debate [3, 6]. This systematic review and meta-analysis aim to assess the impact of wide versus narrow surgical margins on local recurrence rates across the different grades of phyllodes tumors. This protocol has been developed in accordance with the Preferred Reporting Items for Systematic Reviews and Meta-Analysis Protocols (PRISMA-P) checklist [7].

## Methods

### Objectives

This systematic review and meta-analysis aims to evaluate the comparative effect of wide versus narrow surgical margins on local recurrence rates in patients with phyllodes tumors of the breast, stratified by tumor grade.

Specifically, the objectives are the following:

1. To systematically synthesize and meta-analyze the local recurrence rates after surgical excision of phyllodes tumors, comparing outcomes between wide and narrow histopathologically confirmed surgical margins.
2. To stratify the comparative analysis of local recurrence rates by phyllodes tumor grade (benign, borderline, and malignant).
3. To assess and synthesize evidence regarding re-excision rates associated with different surgical margin widths.
4. To explore the reported use of adjuvant treatments and their association with local recurrence across different margin groups.
5. To identify and summarize adverse events reported in studies comparing wide versus narrow surgical margins for phyllodes tumors.

## Study design

This systematic review and meta-analysis will be conducted according to the recommendations of the Cochrane Collaboration [8] and is registered with PROSPERO (link here) [9, 10]. The review will incorporate observational studies (cohort, case–control, and case series with 10 or more patients) and clinical trials reporting local recurrence of phyllodes tumors after surgery with specified margin widths.

## Eligibility criteria

We will consider all studies of surgical excision of phyllodes tumors comparing different width excision margins.

## Inclusion criteria

- Studies involving patients diagnosed with benign, borderline, or malignant phyllodes tumors of the breast.
- Studies reporting local recurrence rates after surgical excision with reported margin widths (negative, positive, ≥ 1 mm, ≥ 2 mm, ≥ 10 mm).
- Observational studies (cohort, case–control, cross-sectional, case series) and randomized controlled trials (RCTs).

## Exclusion criteria

1. Studies where margins were not histopathologically confirmed post-operatively.
2. Studies of other types of breast tumors not including phyllodes tumors.
3. Case reports, case series with fewer than ten patients, and literature reviews.

## Information sources and search strategy

A comprehensive search strategy has been constructed together with an experienced information specialist and will be reported in accordance with the Preferred Reporting Items for Systematic reviews and Meta-Analyses literature search extension (PRISMA-S) checklist to ensure reproducibility. The search will be conducted in electronic bibliographic databases from their respective inception, including MEDLINE, EMBASE, Web of Science, Google Scholar, and Cochrane Library. Gray literature will be searched via OpenGrey, clinical trial registries (e.g., ClinicalTrials.gov and WHO ICTRP), and dissertation databases. Reference lists of included studies will be screened for additional relevant literature and a hand-search will be conducted to identify relevant abstracts from relevant professional society meetings and conference proceedings. Finally, relevant unpublished studies will be identified through co-authorship or after personal contacts with authors. No restrictions will be imposed on publication status or language (translations will be undertaken if a screened title in languages other than English deems the paper possibly pertinent). The search strategy will incorporate both medical subject headings (MeSH) and keywords related to phyllodes tumors, local recurrence, and surgical margins.

## Study selection

All search results will be uploaded to the web-based software platform Covidence (Melbourne, Australia). In Covidence, titles and abstracts of identified studies will be independently screened by two reviewers. If an abstract is absent, the full text will be reviewed. Full-text screening of potentially relevant studies will be performed by two reviewers to determine final inclusion based on predefined eligibility criteria. Discrepancies will be resolved through discussion, and a third reviewer will be consulted if necessary. Reviewers will not be blinded to the authors or journals when screening articles.

## Data extraction

Two reviewers will independently extract data from the included studies using a standardized extraction form in Covidence. The following data will be collected:

- Study characteristics: author, year, country, study design, sample size, follow-up duration.
- Patient and tumor characteristics: age, sex, tumor grade (benign, borderline, malignant).
- Intervention details: margin width (positive, negative, < 1 mm, ≥ 1 mm < 2 mm, ≥ 2 mm, < 10 mm, ≥ 10 mm).
- Outcomes: local recurrence rates
- Variables: re-excision rates, follow-up duration, adjuvant treatments, and adverse events.

Missing data will be requested from study authors where necessary.

## Outcome measures

The main outcome measure will be the pooled incidence of local recurrence, defined as the recurrence of phyllodes tumor at the site of initial excision (ipsilateral breast) and stratified by margin width and tumor grade (benign, borderline, malignant). Depending on available data reported in different studies, local recurrence rates may be assessed as either time-to-event (hazard ratio (HR)) or as odds-ratio for different margin widths at fixed time points.

## Risk of bias assessment

The quality of individual studies will be assessed using the Newcastle–Ottawa Scale (NOS) for observational studies.

The Risk Of Bias In Non-randomized Studies of Interventions (ROBINS-I) tool will be used to assess the risk of bias in cohort and case–control studies and the Cochrane Risk of Bias Tool 2 for RCTs. Both tools are available at https://www.riskofbias.info/. RCTs and observational studies will be pooled separately.

Two independent reviewers will conduct the assessment, and disagreements will be resolved through discussion or consultation with a third reviewer.

## Data synthesis and analysis

Meta-analysis will be conducted using a random-effects model if sufficient homogeneity exists between studies. Local recurrence rates will be compared between groups with different margin widths using HRs and odds ratios (ORs) with 95% confidence intervals (CIs). Heterogeneity will be assessed using the I^2^ statistic, with *I*^2^ > 50% indicating substantial heterogeneity. Subgroup analyses will be performed based on tumor grade (benign, borderline, malignant) and margin width (positive vs negative, < 1 mm vs ≥ 1 mm, < 2 mm vs ≥ 2 mm, < 10 mm vs ≥ 10 mm) and surgical method (breast-conserving vs mastectomy). Analyses will be performed in the web-based software platform Review Manager (RevMan) Version 5.4 (the Cochrane Collaboration. Available at revman.cochrane.org).

RCTs and observational studies will be pooled separately. Sensitivity analyses will be performed excluding studies with high risk of bias.

Where meta-analysis is not feasible, a narrative synthesis of findings will be provided. This will summarize the key characteristics of the studies included (i.e., types of studies, setting, number of participants, and methods of analysis); the methodological quality of studies included, estimates reported, as well as size and direction of effects.

## Meta-bias assessment

Visual assessment of the funnel plot and the Egger’s statistic will be used to assess for both the presence and statistical significance of publication bias across studies, when ten or more studies are included in a meta-analysis [8].

## Reporting

The reporting of this study will follow the guidelines of the Preferred Reporting Items for Systematic reviews and Meta-Analyses (PRISMA 2020) statement [11]. We will follow the “Meta-analysis of Observational Studies in Epidemiology” (MOOSE) guidelines while performing quantitative synthesis and reporting of the observational studies [12]. Details on the search strategy, study selection process, risk of bias assessment, and data synthesis will be thoroughly documented. The results of the review will be submitted for publication in a peer-reviewed journal and presented at relevant academic conferences.

## Discussion

To support future clinical trials, this systematic review aims to map the current landscape of available evidence, synthesized according to gold-standard methods with the latest available data. This review might help guide clinicians in a multi-disciplinary approach to feasible surgical margins in patients with phyllodes tumors by pooling all available evidence. Given that previous systematic reviews that have been published on this topic have some methodological concerns, we aim to provide a more thorough, transparent and granular review. Our results may also be helpful in designing a future RCT, specifically in regards of what might be appropriate margins to select as intervention visavi control arm. Additionally, this review might also be helpful in determining appropriate sample size and power calculation in a future clinical trial. We believe that in lieu of well-designed, prospective RCTs, there is an ethical need for an evidence-based approach to synthesize current available real-world data to optimize surgical treatment. Ultimately, these findings will provide a robust base to update clinical guidelines with new evidence-based recommendations and will establish a strong research base for future studies in this subject field.

## Supplementary Information

Additional file 1: PRISMA-P+checklistR1.

Additional file 2: Proposed search strategy.

## Data Availability

Not applicable.
