# Supplementary material for: Local recurrence of phyllodes tumors after surgery with wide compared to narrow margins: study protocol for a systematic review and meta-analysis
Source: Syst Rev. 2025 Jul 8;14:142. doi: 10.1186/s13643-025-02904-1 (PMC12235997; doi:10.1186/s13643-025-02904-1)
Supplement: Supplementary file 2 — Additional file 2: Proposed search strategy. [file 13643_2025_2904_MOESM2_ESM.docx]

This document outlines a proposed search strategy for a systematic review and meta-analysis on the local recurrence of phyllodes tumors after surgery with wide compared to narrow margins. This strategy was developed in April 2025 by Carl Sars from the Department of Molecular Medicine and Surgery, in collaboration with librarians Emma-Lotta Säätelä and AnnaMia Eborn Martinovic from the Karolinska Institutet University Library.

The literature search will be performed in the following databases: Medline (Ovid), Embase (embase.com), Cochrane Library (Wiley), Web of Science Core Collection (Clarivate Analytics), CINAHL (EBSCOhost), ProQuest Dissertations & Theses Global: The Sciences and Engineering Collection (ProQuest), LILACS, and Google Scholar. Additionally, trial registries including ClinicalTrials.gov and the International Clinical Trials Registry Platform (ICTRP) will be searched.

The search strategy was developed in Medline (Ovid). For each search concept, Medical Subject Headings (MeSH-terms) and free text terms will be identified. The search will then be translated, in part using Polyglot Search Translator. No language restriction will be applied, and databases will be searched from inception. The strategies will be proofread by another librarian prior to execution. De-duplication will be done using Covidence. The full search strategies for all databases will be available in an appendix.

References:

1. Clark JM, Sanders S, Carter M, Honeyman D, Cleo G, Auld Y, Booth D, Condron P, Dalais C, Bateup S, Linthwaite B, May N, Munn J, Ramsay L, Rickett K, Rutter C, Smith A, Sondergeld P, Wallin M, Jones M, Beller E. (2020) Improving the translation of search strategies using the Polyglot Search Translator: a randomized controlled trial. *Journal of the Medical Library Association: JMLA*. 108(2):195-207. doi: 10.5195/jmla.2020.834.
2. Covidence systematic review software, Veritas Health Innovation, Melbourne, Australia. Available at www.covidence.org.

**Proposed Search Strategies**

**1. Medline (Ovid)**

*Interface:* Ovid MEDLINE(R) ALL content coverage from 1946
*Comment:* In Ovid, two or more words are automatically searched as phrases; i.e. no quotation marks are needed.
*Field labels:*
* exp/ = exploded MeSH term
* / = non exploded MeSH term
* .ti,ab,kf. = title, abstract and author keywords
* adjx = within x words, regardless of order
* * = truncation of word for alternate endings
* ? = 0-1 letter/number
* # = 1 letter/number

| **#** | **Searches** |
| --- | --- |
| 1 | Phyllodes tumor/ |
| 2 | (phyllo?d* or mu?ller? tumo* or pseudosarcomatous fibroadenoma* or giant intracanalicular fibroadenoma* or brodie* serocystic* or cystosarcoma*).ti,ab,kf. |
| 3 | 1 or 2 |
| 4 | Recurrence/ |
| 5 | Neoplasm Recurrence, Local/ |
| 6 | (recur* or relaps* or recrudescen*).ti,ab,kf. |
| 7 | Margins of Excision/ |
| 8 | margin?.ti,ab,kf. |
| 9 | su.fs. |
| 10 | exp Surgical Procedures, Operative/ |
| 11 | (surg* or operative or operation).ti,ab,kf. |
| 12 | or/4-11 |
| 13 | 3 and 12 |

**2. Embase (embase.com)**

*Interface:* embase.com content coverage from 1947
*Comment:* Emtree is the controlled vocabulary in Embase.
*Field labels:*
* /exp = exploded Emtree term
* /de = non exploded Emtree term
* ti,ab,kw = title, abstract and author keywords
* NEAR/x = within x words, regardless of order
* * = truncation of word for alternate endings
* $ = 0-1 letter/number
* ? = 1 letter/number

| **No.** | **Query** |
| --- | --- |
| #12 | #3 AND #11 |
| #11 | #4 OR #5 OR #6 OR #7 OR #8 OR #9 OR #10 |
| #10 | surg*:ti,ab,kw OR operative:ti,ab,kw OR operation:ti,ab,kw |
| #9 | surgery:lnk |
| #8 | 'surgery'/exp |
| #7 | margin:ti,ab,kw OR margin?:ti,ab,kw |
| #6 | 'surgical margin'/de |
| #5 | recur*:ti,ab,kw OR relaps*:ti,ab,kw OR recrudescen*:ti,ab,kw |
| #4 | 'recurrent disease'/de OR 'tumor recurrence'/de OR 'recurrence risk'/de OR 'cancer recurrence'/exp OR 'recurrence free survival'/exp |
| #3 | #1 OR #2 |
| #2 | phyllo<span class="math-inline">d\*:ti,ab,kw OR 'mu\$ller\$ tumo\*':ti,ab,kw OR 'pseudosarcomatous fibroadenoma\*':ti,ab,kw OR 'giant intracanalicular fibroadenoma\*':ti,ab,kw OR 'brodie\* serocystic\*':ti,ab,kw OR cystosarcoma\*:ti,ab,kw \ |
| \ | #1 \ |

**3. Cochrane Library (Wiley)**

*Interface:* Wiley content coverage: -Cochrane Database of Systematic Reviews - April 1996, Central Trials - Current content July 1998
*Field labels:*
* mh = exploded MeSH term
* mh ^= non exploded MeSH term
* ti,ab,kw = title, abstract and author keywords
* NEAR/x = within x words, regardless of order
* NEXT = used for truncated phrases
* NEXT/x = fixed word order
* * = truncation of word for alternate endings
* ? = 0-1 letter/number

**ID Search**

#1 [mh ^"Phyllodes tumor"]
#2 (phyllo?d*:ti,ab,kw OR (mu?ller? NEXT tumo*):ti,ab,kw OR ("pseudosarcomatous" NEXT fibroadenoma*):ti,ab,kw OR ("giant intracanalicular" NEXT fibroadenoma*):ti,ab,kw OR (brodie* NEXT serocystic*):ti,ab,kw OR cystosarcoma*:ti,ab,kw)
#3 #1 or #2

**4. Web of Science Core Collection (Clarivate Analytics)**

*Interface:* Clarivate Analytics
*Editions and content coverage years:* A&HCI - 1975, ESCI - 2019, SCI-EXPANDED - 1945, SSCI - 1945
*Note:* The Exact search -function will be used for all the searches.
*Field labels:*
* TS/Topic = title, abstract, author keywords and Keywords Plus
* TI= title
* AB = abstract
* AK = author keywords
* NEAR/x = within x words, regardless of order
* * = truncation of word for alternate endings
* $ = 0-1 letter/number
* ? = 1 letter/number

**# Search Query**

1 TS=(phyllo$d* OR "muller$ tumo*" OR "mueller$ tumo*" OR "pseudosarcomatous fibroadenoma*" OR "giant intracanalicular fibroadenoma*" OR "brodie* serocystic*" OR cystosarcoma* )
2 TS=(recur* OR relaps* OR recrudescen* )
3 TS=margin$
4 TS=(surg* OR operative OR operation )
5 #4 OR #3 OR #2
6 #5 AND #1

**5. CINAHL (EBSCOhost)**

*Interface:* EBSCOhost - content coverage from 1981 *Note:* Sometimes "quotation marks" are needed for single search terms to avoid automatic term mapping (lemmatization). *Field labels:* * MH+ = exploded Cinahl Heading * MH = non exploded Cinahl Heading * TI = title * AB = abstract * Nx = within x words, regardless of order * * = truncation of word for alternate endings * # = 0-1 letter/number * ? = 1 letter/number

| **#** | **Query** | **Limiters/Expanders** |
| --- | --- | --- |
| S9 | S1 AND S8 | Expanders - Apply equivalent subjects, Search modes - Find all my search terms |
| S8 | S2 OR S3 OR S4 OR S5 OR S6 OR S7 | Expanders - Apply equivalent subjects, Search modes - Find all my search terms |
| S7 | MW surg* | Expanders - Apply equivalent subjects, Search modes - Find all my search terms |
| S6 | XB (surg* or operative or operation) | Expanders - Apply equivalent subjects, Search modes - Find all my search terms |
| S5 | XB margin# | Expanders - Apply equivalent subjects, Search modes - Find all my search terms |
| S4 | (MH "Surgery, Operative+") | Expanders - Apply equivalent subjects, Search modes - Find all my search terms |
| S3 | XB (recur* OR relaps* OR recrudescen*) | Expanders - Apply equivalent subjects, Search modes - Find all my search terms |
| S2 | (MH "Recurrence+") OR (MH "Neoplasm Recurrence, Local") | Expanders - Apply equivalent subjects, Search modes - Find all my search terms |
| S1 | XB ((phyllo#d* OR "mu#ller# tumo*" OR "pseudosarcomatous fibroadenoma*" OR "giant intracanalicular fibroadenoma*" OR "brodie* serocystic*" OR cystosarcoma*)) | Expanders - Apply equivalent subjects, Search modes - Find all my search terms |

**6. ProQuest Dissertations & Theses Global: The Sciences and Engineering Collection**

*Interface:* ProQuest
*Content coverage years:* ProQuest dissertations & theses from 1673
*Field labels:*
* noft = anywhere except full text

| **Query** | **Other actions** |
| --- | --- |
| noft((phyllo?d* OR "mu?ller? tumo*" OR "pseudosarcomatous fibroadenoma*" OR "giant intracanalicular fibroadenoma*" OR "brodie* serocystic*" OR cystosarcoma*)) | Limited by: Manuscript type: Doctoral dissertations |

**7. LILACS**

*Website:* <https://lilacs.bvsalud.org/en/>

| **Field** | **Query** |
| --- | --- |
| Title, abstract, subject: | phyllodes OR phylloides OR phylloid AND db:("LILACS") AND instance:"lilacsplus" |

**8. Google Scholar**

*Interface:* Harzing, A.W. (2007) *Publish or Perish*, available from <https://harzing.com/resources/publish-or-perish>
*Search syntax:*
* Character limit for search strings is 256 characters
* Boolean: AND is represented by a space and OR is represented by a vertical line [ | ]
* Phrase searching with quotation marks
* Does not recognize truncation symbols - Plural, genitive and other word forms will be searched automatically
* Field search: Keywords - searches the fulltext of scholarly material, including citation and abstract

| **Keywords:** | **Include:** | **Relevance ranked:** |
| --- | --- | --- |
| phyllodes\ | phylloides\ | phylloid recurrence\ |

**9. ClinicalTrials.gov**

*Website:* clinicaltrials.gov
*Searched in:* Other terms

| **Other terms:** |
| --- |
| phyllodes OR phylloides OR phylloid |

**10. International Clinical Trials Registry Platform – ICTRP**

*Website:* <https://trialsearch.who.int/>

| **Query:** |
| --- |
| phyllodes OR phylloides OR phylloid |
